# Identification of a Broad Bean Wilt Virus 2 (BBWV2) Isolate (BBWV2-SP) from *Spinacia oleracea* L.

**DOI:** 10.3390/ijms26135946

**Published:** 2025-06-20

**Authors:** Xu Zhao, Zhiyuan Liu, Hongbing She, Zhaosheng Xu, Helong Zhang, Wujun Gao, Wei Qian

**Affiliations:** 1 State Key Laboratory of Vegetable Biobreeding, Institute of Vegetables and Flowers, Chinese Academy of Agricultural Sciences, Beijing 100081, China; zhaoxuhtu@163.com (X.Z.); liuzhiyuan01@caas.cn (Z.L.); shehongbing@caas.cn (H.S.); xuzhaosheng@caas.cn (Z.X.); zhanghelong@caas.cn (H.Z.); 2 College of Life Sciences, Henan Normal University, Xinxiang 453007, China; 3 Zhongyuan Research Center, Chinese Academy of Agricultural Sciences, Xinxiang 453000, China

**Keywords:** spinach, broad bean wilt virus 2, RNA-seq, RT-PCR

## Abstract

Spinach (*Spinacia oleracea* L.) is an important leafy vegetable but is vulnerable to viral infections that significantly affect its quality and yield. In this study, we identified virus-infected spinach exhibiting typical symptoms with yellowing, wrinkling, and mottling in Beijing. But conventional RT-PCR screening for twelve common plant viruses yielded negative results. Then, using transcriptome sequencing along with a de novo assembly approach, we obtained the complete viral genome, which consists of RNA1 (5916 nucleotides) and RNA2 (3576 nucleotides). BLASTN analysis against the NCBI viral genome database revealed high homology with broad bean wilt virus 2 (BBWV2), leading us to designate this isolate as BBWV2-SP (GenBank accession numbers PV102464 and PV102465). Phylogenetic analysis indicated that BBWV2-SP shares 96.69% nucleotide sequence identity with a Liaoning isolate from *Chenopodium album* MN786955 and clusters within the Chinese evolutionary lineage. We developed primers targeting the conserved region of the RNA2 coat protein, amplifying a 478-base-pair product. All symptomatic spinach samples tested positive, while asymptomatic controls remained negative, confirming the causal relationship between BBWV2-SP and the observed disease symptoms. This study provides the complete genome assembly of the spinach isolate BBWV2-SP and establishes a molecular detection protocol for BBWV2 in spinach. These findings offer essential technical support for field monitoring, epidemiological surveillance, and disease control strategies, while also enhancing our understanding of BBWV2′s genetic diversity and mechanisms of pathogenicity.

## 1. Introduction

Spinach (*Spinacia oleracea* L.), belonging to the *Amaranthaceae* family, *Chenopodioideae* subfamily, typically grown for its edible dark-green leaves, is cultivated in over 60 countries worldwide. The area of spinach production has been increasing year by year. According to data from the Food and Agriculture Organization, the area of spinach production reached 735,639 hectares in 2023 (https://www.fao.org/faostat (accessed on 1 February 2025)). Spinach contains high amounts of minerals (Calcium, Phosphorus, Iron, Potassium), vitamins and antioxidant molecules [1], and most nutrients are concentrated in the leaves [2]. 

As with most agricultural commodities, diseases impose significant production constraints affecting both the yield and overall quality of spinach. There have been 12 naturally occurring virus diseases reported in spinach [3], and cucumber mosaic virus (CMV), beet western yellows virus (BWYV) and turnip mosaic (TuMV) are three of the more common [4].

Historically, the most significant is CMV, which is capable of infecting well over 1000 plant species, causing significant economic losses in various crops [5]. CMV is a positive-sense RNA plant virus with a genome consisting of three single-stranded RNA molecules, designated RNA1, RNA2 and RNA3 [6]. In spinach, CMV causes a disease known as ‘Spinach Blight’. Symptoms vary greatly according to cultivar, plant age, temperature, and virus strain, including chlorotic mottling, narrow, severely wrinkled with veinal distortions, and inward rolling of margins [4]. According to a global survey spanning 28 countries and regions [7], TuMV was identified as the second most prevalent viral pathogen in field-grown vegetables, following CMV. It has worldwide distribution, including temperate and tropical regions of Africa, Asia, Oceania, Europe and North and South America [8]. Disease symptoms associated with TuMV infection depend upon the TuMV strain, host plant and environmental conditions. Symptoms are diverse but often include vein clearing, chlorotic mottling, leaf distortion, mosaic, necrosis, plant stunting and plant death [9]. BWYV is the most well-known member of the genus *Polerovirus* in the family *Luteoviridae* [10]. The extensive host range of BWYV includes over 150 plant species from 23 dicotyledonous families. Symptoms of spinach yellowing plants in the field vary in severity depending on the variety but mainly consist of the lower leaves becoming yellow, thick and rough, and the edges of the leaves becoming wavy [11]. Small reddish-brown necrotic spots sometimes appear in the yellowed areas. Necrosis often occurs from the tips of the leaves, and, in some cases, the entire plant dies.

Despite the well-documented threats posed by these known naturally occurring infectious viruses, undetected viruses in spinach continue to challenge spinach agricultural systems. Among these, broad bean wilt virus (BBWV) represents a new risk. BBWV is a devastating pathogen of many economically important horticultural and ornamental crops. BBWV is a member of the genus *Fabavirus* in the family *Secoviridae* [12]. It is spread through aphids, mostly *Aphis gossypii* and *Myzus persicae*, with infection rates of 60–90% in a nonpersistent manner and has a wide host range [13,14]. In serological and molecular studies, BBWV isolates are classified into two species, broad bean wilt virus 1 (BBWV1) and broad bean wilt virus 2 (BBWV2) [15,16]. Although they show similar genome structures and functions, the nucleotide (nt) sequence identity between the two BBWV species was limited (39–67%). The genome is composed of two single-stranded positive-sense RNA molecules, RNA1 and RNA2, that are encapsidated separately into icosahedral virions [17]. While BBWV1 has been reported mainly in Europe and North America, BBWV2 has a worldwide distribution. In China, BBWV1 has not been detected yet, but disease incidences caused by BBWV2 were reported in a variety of plants, such as marigold, balsam, *Mirabilis jalapa*, sesame and faba bean in the past few years [18,19,20,21,22].

Nearly 47% of the world′s plant disease epidemics are caused by a virus [23]. These diseases often occur over the entire field, and it is difficult to distinguish from nutrient deficiency or soil disease based on the symptoms alone [24]. The best practice to prevent crops from a viral infection is to diagnose the infected plant and destroy it. Reverse Transcription–Polymerase Chain Reaction (RT-PCR), with high specificity and extremely high sensitivity, is increasingly used in plant RNA virus detection. As we face the challenges of emerging and re-emerging diseases and the intentional and unintentional introduction of plant viruses into crops, the speed and accuracy of detection become critical. Many RNA viruses have been detected using RT-PCR [25,26,27,28]. However, the prerequisite of having known sequences to select and synthesize suitable primers limits its application to uncharacterized viruses. When the virus that infects plants is not a common naturally occurring virus, it is difficult to detect the virus using RT-PCR. But high-throughput sequencing (HTS) can discover novel viruses without any prior knowledge regarding viral pathogens and helps in the detection process in a nonspecific fashion. *Cyclamen persicum,* which exhibited typical virus-associated symptoms such as leaf deformation, yellowing, leaf mottling, and flower breaking did not reveal the presence of any virus via RT-PCR-based assaying. But when HTS was adopted, which fortunately does not require any prior information regarding the viral sequence, it revealed the presence of fig mosaic emaravirus (FMV) [29]. A very large number of viruses were identified in new hosts by HTS, such as fig mosaic emaravirus was found in a non-fig host, *C. persicum* [30].

Recently, diseased spinach was found in Beijing; the leaves of spinach showed different degrees of chlorotic mottling, with narrow, severely wrinkled and inward rolling margins. This unknown virus may have a severe impact on spinach production, so identifying the virus species is urgent. We attempted to detect the molecular markers of 12 common plant viruses using RT-PCR, but no viruses were matched. Through HTS analysis, genome assembly of the unknown virus, and BLASTN searches against the NCBI nonredundant database, the viral sequences were found to be homologous to BBWV2.

## 2. Results

### 2.1. Viral Disease Phenotype Observation

**Figure 1:**
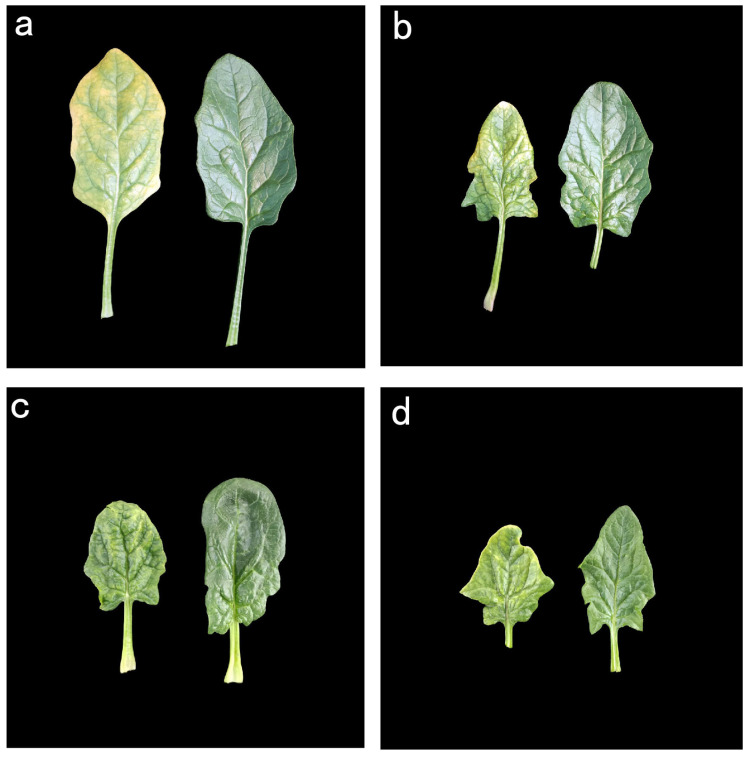
Leaf samples were collected from spinach plants displaying major virus-like symptoms: (**a**) yellowing, (**b**) narrow, (**c**) severely wrinkled, (**d**) chlorotic mottling. The left side shows the infected leaf, and the right side shows the healthy leaf.

Leaf samples exhibiting virus-like symptoms from different disease-level spinach were collected in Beijing. After observation, the phenotype mainly included yellowing, narrowing, severe wrinkling, and chlorotic mottling (Figure 1).

### 2.2. Virus Detection by RT-PCR

The eight collected samples, numbered from 1 to 8, were tested via RT-PCR, with samples 1–4 asymptomatic and 5–8 symptomatic. We detected 12 common viruses in spinach via RT-PCR [3]. All primers have been reported for the detection of CMV [31], TuMV [32], BWYV [33], tobacco mosaic virus (TMV) [34], tomato mosaic virus (ToMV) [35], tobacco etch virus (TEV) [36], tobacco rattle virus (TRV) [37], tomato spotted wilt virus (TSWV) [37], tomato ringspot virus (ToRSV) [38], tomato black ring virus (TBRV) [39], tomato yellow leaf curl virus (TYLCV) [40], tomato brown rugose fruit virus (ToBRFV) [41]. The results showed that CMV, TuMV, BWYV, TMV, ToMV, TRV, TSWV, ToRSV, TBRV, ToBRF and TYLCV had no bands (Figure S1). TEV had many mixed bands, and both symptomatic and asymptomatic samples had bands. In summary, it was determined that the 12 viruses detected were not the culprits of spinach virus disease.

### 2.3. Identification of Viral Genome-Derived Contigs by Spinach Reads

Raw reads were filtered using fastp (v0.23.3) with the parameter “−q 20” to remove low-quality reads, contaminated joints and those with high unknown base N content. Clean reads were aligned to the SILVA ribosomal rRNA database to eliminate the reads derived from rRNA. Thus, 3,908,556 reads were removed to obtain 149,120,430 clean reads mixed from three symptomatic samples. Clean reads were aligned to the spinach reference genome Monoe-Viroflay [42] using HISAT2 (v2.8.2) and sorted using samtools v1.15 with default parameters, to remove host reads. All reads unaligned to spinach genome were assembled by Trinity and Megahit software, resulting in 604 contigs. After filtering, 46 contigs were chosen as candidate viral contigs. BLASTN searching based on the contigs showed that two contigs with lengths of 6017 and 3839 nt have high homology to RNA1 and RNA2 of BBWV2 (GenBank accession numbers OP783967 and MN786955.1), which belongs to the species *Fabavirus betaviciae* in the family *Secoviridae*.

**Figure 2:**
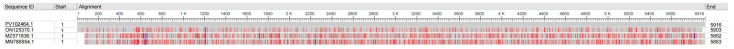
Analysis of BBWV2-SP RNA1 genome integrity and conservation. Red lines represent bases different from BBWV2-SP and blue lines represent the presence of base deletions.

**Figure 3:**
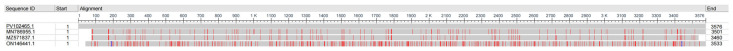
Analysis of BBWV2-SP RNA2 genome integrity and conservation. Red lines represent bases different from BBWV2-SP and blue lines represent the presence of base deletions.

In order to determine the integrity of the potential viral genome identified in spinach, the contigs obtained in the above steps were compared with the NCBI viral genome database. More than 100 virus isolates were highly homologous to candidate contigs, from which 90–100 of the closest viral genomes were extracted. Mega software was used to perform multiple alignments of different virus isolates to identify the conserved regions at both ends of the viral genome (Figure 2 and Figure 3). The results showed that candidate contigs completely covered the conserved regions at both ends. Candidate contigs after removing redundant sequences at the 5′ and 3′ ends compared to other contigs were accepted as the full-length genome of BBWV2 RNA1 (5916 nt) and RNA2 (3576 nt). The sequence was uploaded to NCBI (Genbank accession number PV102464 and PV102465). Therefore, in this study, only viruses derived from BBWV2 were considered, and these candidates were named as BBWV2-SP according to the sample species infected by virus.

### 2.4. Evolutionary Characteristics and Genome Structure of BBWV2-SP

**Figure 4:**
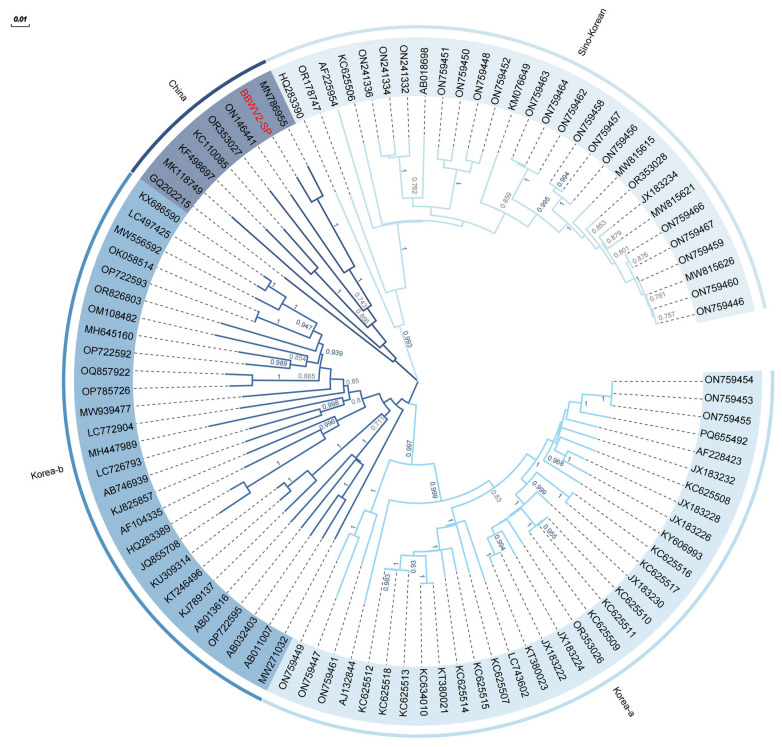
Evolutionary relationship based on BBWV2-SP RNA2 nucleotide sequences. Branch length scale is 0.01.

To gain a deeper understanding of the evolutionary genetic characteristics of BBWV2-SP in the BBWV2 species, 98 non-recombinant BBWV2 samples with full-length and near-full-length genomes identified in the NCBI database were used as research objects to analyze the phylogenetic relationships among BBWV2 isolates. A maximum likelihood (ML) tree based on the RNA2 sequence was constructed using Mega X (Figure 4). The BBWV2 isolates clearly clustered into four subgroups, named Korea-a, Korea-b, China, Sino-Korea, according to the region where isolates were collected. Most isolates in subgroups Korea-a and Korea-b were collected in Korea. All isolates in subgroup China were collected in China. The Sino-Korea subgroup contains isolates from both China and Korea, exhibiting a peculiar cross-geographical distribution. The BBWV2 identified in this study was classified in subgroup China and showed close phylogenetic relationships with BBWV2-LNSY (MN786955.1). Nucleotide sequence identity reached 96.69%.

**Figure 5:**
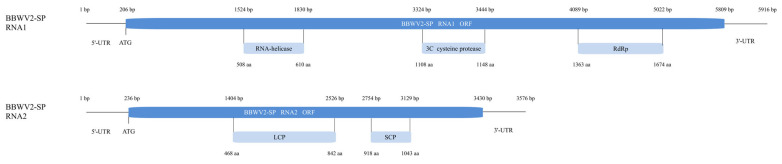
Schematic diagram of genomic structure of BBWV2-SPRNA1 and RNA2.

By comparing BBWV2-SP to the NCBI viral genome database, BBWV2-SP was confirmed as belonging to the species *Fabavirus betaviciae* in the family *Secoviridae*. The size of the BBWV2-SP RNA1 genome was 5916 nucleotides (nt), in which a 5604 bp open reading frame (ORF) located between 206 and 5809 bp encoded an 1867 aa polyprotein. This polyprotein will be processed into several proteins involved in genome replication and expression. Protein structure analysis found that the polyprotein contains conserved domains, including RNA-helicase (between 508 and 610 aa), 3C cysteine protease (between 1108 and 1148 aa), RNA-dependent RNA polymerase (RdRp, between 1363 and 1674 aa). The RNA2 genome was 3576 nt. ORF, which is 3195 bp, located between 236 and 3430 bp, encoded a 1064 aa polyprotein. This polyprotein will be further cleaved into two coat proteins. Protein structure analysis found conserved domains of large coat protein (LCP, between 468 and 842 aa) and small coat protein (SCP, between 918 and 1043 aa) in this polyprotein (Figure 5).

### 2.5. Design and Validation of BBWV2-SP Molecular Primers

**Figure 6:**
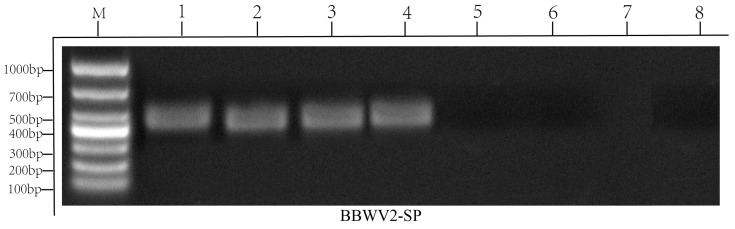
RT-PCR validation for the BBWV2-SP in spinach leaf samples. Lanes 1–4, symptomatic plants. lanes 5–8 asymptomatic plants. Four symptomatic have 478 bp bands.

To confirm the association of BBWV2-SP with the observed disease, sequences of the LCP conserved domain were used to design primers of BBWV2-SP. Four symptomatic (1–4) and four asymptomatic samples (5–6) cDNAs were used to verify primers. Gel electrophoresis results show that four symptomatic samples have 478 bp bands, while four asymptomatic samples have no bands (Figure 6). Validation experiments confirmed the causal relationship between BBWV2-SP and the spinach viral disease. To confirm the specificity of PCR products, Sanger sequencing was performed on four independent symptomatic samples. Sequence alignment demonstrated 100% identity between the sequence of products and the corresponding HTS-assembled genome region (nt 2864–3341 of RNA2, Figure S2).

## 3. Discussion

As a vegetable with leaves as the main edible part, spinach is very susceptible to diseases, such as downy mildew, caused by *Peronospora effusa* (*P. farinosa* f. sp. *spinaciae*) [4]; anthracnose, caused by *Colletotrichum spinaciae* (*C. dematium* f. sp. *spinaciae*) [43]; white rust, caused by *Albugo occidentalis* [4] and other fungal infections [44]; as well as common virus infections, such as CMV, TuMV, BWYV, etc. [24,45,46]. Beyond these known viruses, novel or unreported viral pathogens in spinach could also pose significant threats to production and breeding. Usually, it is difficult to judge the type of viruses by the phenotype of infection. RT-PCR is a commonly used method to quickly detect plant virus species; however, RT-PCR encounters limitations in detecting uncharacterized or emerging viral pathogens, as it relies on pre-existing sequence information for primer design. Using high-throughput sequencing to detect potential viral sequences and assemble their complete genomes can well assist RT-PCR to identify uncommon viruses. In this study, the culprit was not found during the RT-PCR detection of spinach samples with viral symptoms. After transcriptome sequencing and genome assembly, two sequences were found. After being compared with the NCBI viral genome database, they were highly homologous to the two genomes, RNA1 and RNA2, of BBWV2. We named it BBWV2-SP. The assembled genome’s full-length sequence was uploaded to NCBI, and the GeneBank ID is PV102464 and PV102465. At the same time, molecular markers designed with the LCP conserved region of BBWV2-SP RNA2 were verified by RT-PCR, and we found that the corresponding bands could be detected in symptomatic samples, while there was no band in asymptomatic samples, which was associated with viral infestation. Therefore, it can be determined that spinach disease in Beijing is related to BBWV2. However, due to the lack of verification of the Koch postulates, it cannot be concluded that spinach disease is indeed caused by BBWV2, and it is not ruled out that BBWV2 is combined with other viruses under natural conditions. Subsequent experiments will try to construct infectious clones of BBWV2 to provide more data on its pathogenic properties.

BBWV2 is a member of the genus *Fabavirus betaviciae* of the *Comovirinae* subfamily, *Secoviridae* family. BBWV2 is an important pathogen that causes widespread damage in broad beans, peppers, yams and other economically important horticultural and ornamental crops around the world, and it is transmitted by aphids in a nonpersistent manner [47], causing flower and leaf dwarfing; wilting and plant death lead to obvious negative harms. In a nationwide census of major viral diseases in vegetable crops across 31 provinces in China, BBWV2 was detected in 4.82% of samples, ranking among the top four most prevalent viruses infecting vegetable crops. This highlights BBWV2’s status as a dominant viral pathogen in Chinese vegetable production [48].

We assembled BBWV2 RNA1 and RNA2 genomes, with lengths of 5916 and 3576 (nt), respectively, and each RNA segment contains a single ORF, thus being translated into a single polyprotein precursor, which is further processed by proteolytic cleavage to yield functional proteins. BBWV2 RNA1 usually encodes three conserved domains, including RNA-helicase, 3C cysteine protease and RdRp, while RNA2 encodes two conserved domains, including LCP and SCP [49], which is consistent with our results and demonstrates the integrity of our assembled genome. At the same time, according to the evolutionary tree analysis of the BBWV2 RNA2 nucleotide sequence, the BBWV2 isolates are mainly divided into four subgroups. We named these four subgroups Korea-a, Korea-b, China, and Sino-Korea based on the locations reported for each isolate. The BBWV2 identified in this study was classified in subgroup China and showed close phylogenetic relationships with MN786955.1. The phylogenetic analysis of BBWV2 isolates revealed distinct evolutionary patterns characterized by geographical clustering and cross-regional transmission dynamics. The bifurcation of four major clades, with two clades exclusively comprising Korean strains and one clade containing solely Chinese strains, suggests long-term ecological isolation driven by localized host–virus coevolution, geographical barriers, or restricted agricultural trade practices. The fourth hybrid clade, incorporating both Chinese and Korean strains, demonstrates persistent gene flow, recombination or cross-border introduction events.

The most striking of these isolates is the MN786955.1 isolates, named LNSY. LNSY infects *Chenopodium album* L., which has the highest homology with BBWV2-SP and is also a member of the *Amaranthaceae* family like spinach. In BBWV2-SP, LNSY may have some similar sequence variations that enable BBWV2 to infect *Amaranthaceae* plants. *Chenopodium album* L. is one of the main weeds that harm agricultural production, widely distributed throughout China [50]. Considering the extremely high homology, the spinach BBWV2 isolate and LNSY likely derive from a common ancestor or represent recent divergent lineages within the same clade, particularly given their shared host family *Amaranthaceae*. In addition, validation experiments confirmed the causal relationship between BBWV2-SP and the spinach viral disease.

In general, this study identified the spinach virus isolate BBWV2, reported the RNA1 and RNA2 genome sequences of the BBWV2 spinach isolate, and constructed an ML evolutionary tree based on the BBWV2 RNA2 nucleotide sequence. In addition, we designed molecular markers based on the LCP conserved domain of BBWV2-SP that can be used to rapidly detect BBWV2 in spinach and other plants, providing a basis for virus prevention.

## 4. Materials and Methods

### 4.1. Plant Materials

Leaf samples from spinach line (Sp37) displaying virus-like symptoms were collected from Beijing in open fields of the Chinese Academy of Agricultural Sciences (Figure 1). The age of plants is about one month. Samples consisted of fresh leaves from symptomatic and asymptomatic plants. Each collected leaf sample was immediately frozen with liquid nitrogen and stored at −80 °C.

### 4.2. Extraction of Total Nucleic Acids

Total RNA was extracted separately from six samples using the RNAprep Pure Plant Kit (TIANGEN, Beijing, China) according to the manufacturer’s instructions. NanoDrop2000 and 8000 spectrophotometer (Thermo Fisher Scientific, Waltham, MA, USA) was used to evaluate the concentration and purity of the extracted RNA. We used Agilent2100 Bioanalyzer (Agilent Technologies, Santa Clara, CA, USA) to evaluate the integrity of RNA and diluted the RNA to an appropriate concentration before evaluation. RNA concentration should usually be between 100 ng/μL and 1 μg/μL. After the determination of RNA quality, the total RNA was divided into two portions: one portion was used for virus detection by RT-PCR, and the other portion was used for transcriptome sequencing.

### 4.3. Virus Detection by RT-PCR

To detect viruses in spinach leave samples, cDNA was synthesized from total RNA using HiScript III All-in-One RT SuperMix Perfect (Vazyme, Nanjing, China). PCR amplifications were performed using the following program: initial denaturation at 95 °C for 3 min; 35 cycles of denaturation at 95 °C for 30 s, annealing Tm (Table 1) for 30 s, and extension 72 °C for 30 s; and a final extension at 72 °C for 10 min.

### 4.4. Transcriptome Sequencing and Capturing Viral Genomes in Spinach Through RNA Assembly

Total RNA from three symptomatic samples was used as the input for RNA-seq library construction. mRNA was subsequently enriched using oligo (dT) magnetic beads and fragmented into short sequences. First-strand cDNA was synthesized using mRNA as a template, which was followed by second-strand cDNA synthesis. cDNA was then purified, and the resulting products underwent a series of procedures, including end repair, adapter ligation, A-tailing, and PCR amplification, to produce a cDNA library for sequencing. The constructed cDNA library was quality-checked. After passing quality control, the cDNA library was sequenced on the NovaSeq 6000 S4 platform using paired-end sequencing (150 bp).

Raw RNA-seq reads were filtered using fastp (v0.23.3) with the parameter “−q 20” and aligned to the SILVA ribosomal rRNA database to eliminate the reads derived from rRNA. All reads from three symptomatic samples were mixed into clean reads. Clean reads were aligned to the spinach reference genome Monoe-Viroflay using HISAT2 (v2.8.2) and sorted using samtools with default parameters, to remove host reads. All the clean reads were assembled into contigs by Trinity and Megahit software. Contigs longer than 1000 bp were considered to be candidate viral contigs. After a series of quality control and assemble procedures were performed, the candidate contigs were mapped to the NCBI viral database (https://www.ncbi.nlm.nih.gov/genome/viruses (accessed on 10 February 2025)) to identify transcript-related viruses.

### 4.5. Evolutionary Characteristics and Genome Structure of BBWV2-SP

To understand the evolutionary relationship of spinach BBWV2 virus with different BBWV2 isolates from other plants, 98 non-recombinant BBWV2 viruses with full-length and near-full-length genomes were extracted from NCBI according to references. The maximum likelihood (ML) phylogenetic tree of BBWV2-SP and other non-recombinant BBWV2 isolates was constructed using MEGA software (version: 10.2.6) (https://www.megasoftware.net (accessed on 10 February 2025)) [51]. First, the best-fit ML stress model of BBWV2 isolates was calculated using subroutines in MEGA software. The ML tree was based on the best-fit model with 1000 replicates and other default parameters. The proteins encoded by BBWV-SP were preliminarily determined based on known homologous viruses. ORFfinder was also used to identify the coding regions in the BBWV2 genome. The analysis results were combined with the highly homologous BBWV2 virus genome structure, and the BBWV2 genome structure was analyzed using ORFfinder software (https://www.ncbi.nlm.nih.gov/orffinder (accessed on 10 February 2025)).

### 4.6. Design of Molecular Primers Based on the Conserved Sequence of BBWV2 RNA2 Genome

To detect BBWV2 in symptomatic spinach samples, MEGA software was used to perform multiple alignments of different virus isolates to identify the conserved domains in RNA2 genome. Identified conserver region was extracted to design primer assisted by Primer3Plus (https://www.primer3plus.com (accessed on 15 February 2025)). The primer sequence is as follows: F: CTGGGACTAAGCTCACCGTG, R: TTGCGAAACATGAATGCCGG, product size is 478 bp. RT-PCR amplifications were performed using the following program: initial denaturation at 95 °C for 3 min; 35 cycles of denaturation at 95 °C for 30 s, 60 °C for 30 s, and extension 72 °C for 30 s; and a final extension at 72 °C for 10 min. PCR products were sequenced and blast with genome sequence.

## 5. Conclusions

This study identified BBWV2 as a novel pathogen causing viral disease in spinach (*Spinacia oleracea* L.) in Beijing. Through HTS and de novo genome assembly, the complete RNA1 (5916 nt) and RNA2 (3576 nt) sequences of the spinach isolate BBWV2-SP were characterized, showing high homology (96.69%) with a *Chenopodium album* isolate from Liaoning (MN786955) and clustering within the China phylogenetic lineage of BBWV2. Primers targeting the RNA2 LCP conserved domain were developed and validated, enabling reliable detection of BBWV2-SP in symptomatic spinach samples. These findings expand our understanding of BBWV2’s host range and genetic diversity, while the established molecular detection protocol provides essential tools for field surveillance and disease management in spinach production. This discovery highlights the emerging threat of BBWV2 to vegetable crops and underscores the utility of high-throughput sequencing in identifying uncharacterized viral pathogens, facilitating proactive strategies for viral disease control.

## Figures and Tables

**Table 1:** Specific primers for identification of pathogenic species of spinach virus diseases by RT-PCR.

Virus	Primer	Primer Sequence (5′ to 3′)	Tm/°C
CMV	CMVF	TAATTACAGGCCCTTACCCGC	58
	CMVR	TGAGTGGGCAGAGTCGAGTC	
TuMV	TuMVF	CAAGCAATCTTTGAGGATTATG	54
	TuMVR	TATTTCCCATAAGCGAGAATA	
BWYV	BWYVF	CGAATCTTGAACACAGCAGAG	55
	BWYVR	TGTGGG ATCTTGAAGGATAGG	
TMV	TMVF	TTCTTGTCATCAGCGTGGGCCGA	55
	TMVR	AAGTTGCAGGACCAGAGGTCCA	
ToMV	ToMVF	CCGGATCCATGTCTTACTCAATCAC	52
	ToMVR	GTAAGCTTGTTAACTGGGCCCCAACCGGGGGT	
TEV	TEVF	TGATGGATGGTGAGGAG	47
	TEVR	GTGCCGTTCAGTGTCTT	
TRV	TRVF	ATGGGTGACATGTACGATG	54
	TRVR	GAGGCGTCATCGAATTTGT	
TSWV	TSWVF	ATCGGATCCATGTCTAAGGTTAAGCTCAC	55
	TSWVR	ATCCTCGAGTTAAGCAAGTTCTGTGAGTTTTGC	
ToRSV	ToRSVF	GATGTCCTCCATTTGTTTCGCC	60
	ToRSVR	GGAATGTGTCTCCGTCGTTAA	
TBRV	TBRVF	GCAACTAGTGCGAGTGGTAG	58
	TBRVR	CATAAAATTGGAAGCCATCATG	
TYLCV	TYLCVF	AGTCTGAGGCTGTAATGTCGTCC	62
	TYLCVR	CTGTTCGCAAGTATCAATCAAGGT	
ToBRFV	ToBRFV591R	GACAGGTGAATGGAATTTGCCAGATAATTG	55
	ToBRFV591F	AGACATATTTAATACGAATCTGAATCGGCG	

## Data Availability

All data are contained within the article and Supplementary Materials.

## Supplementary Materials

The following supporting information can be downloaded at: https://www.mdpi.com/article/10.3390/ijms26135946/s1.
