# Supplementary figures and images for: Identification of a Broad Bean Wilt Virus 2 (BBWV2) Isolate (BBWV2-SP) from Spinacia oleracea L
Source: Int J Mol Sci. 2025 Jun 20;26(13):5946. doi: 10.3390/ijms26135946 (PMC12249781; doi:10.3390/ijms26135946)

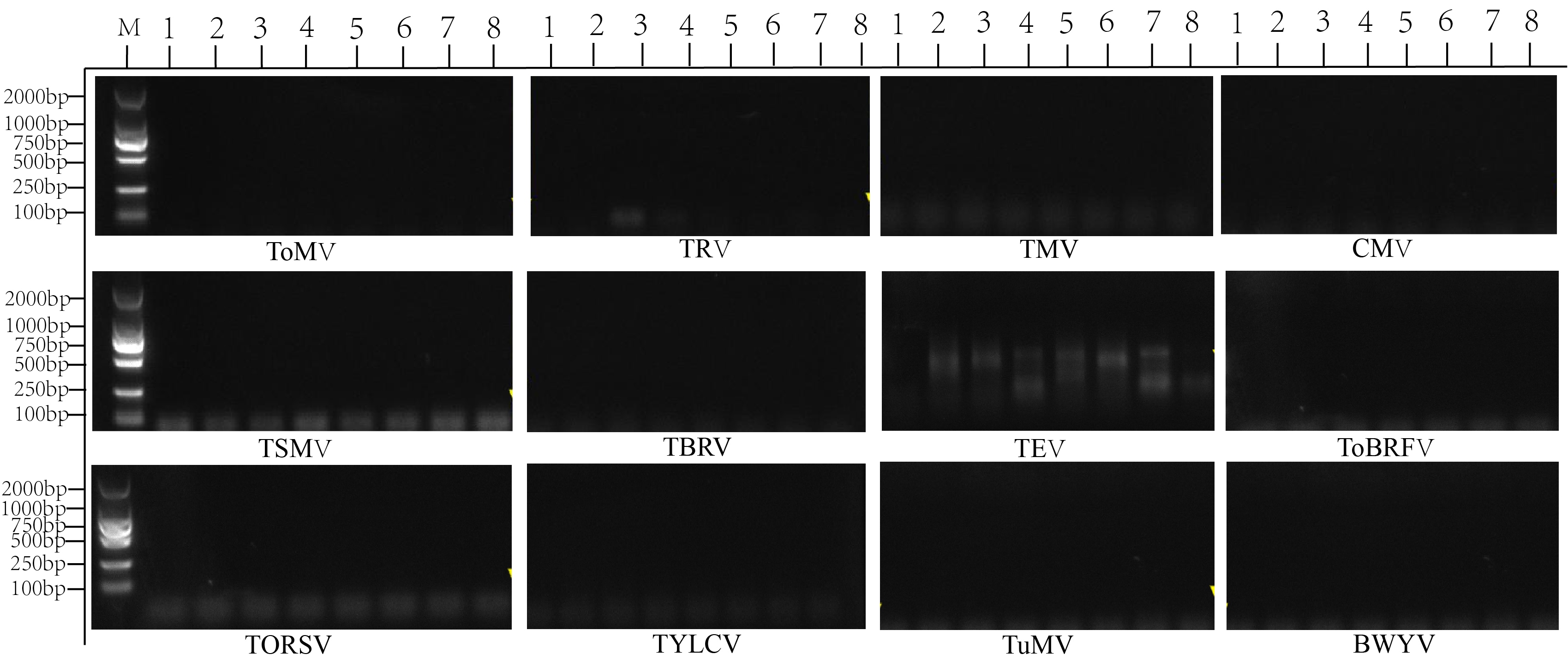

Supplement: Supplementary file 1 [file ijms-26-05946-s001.zip › Figure S1. RT-PCR validation for the presence of thirteen viruses in spinach leaf samples..jpg]

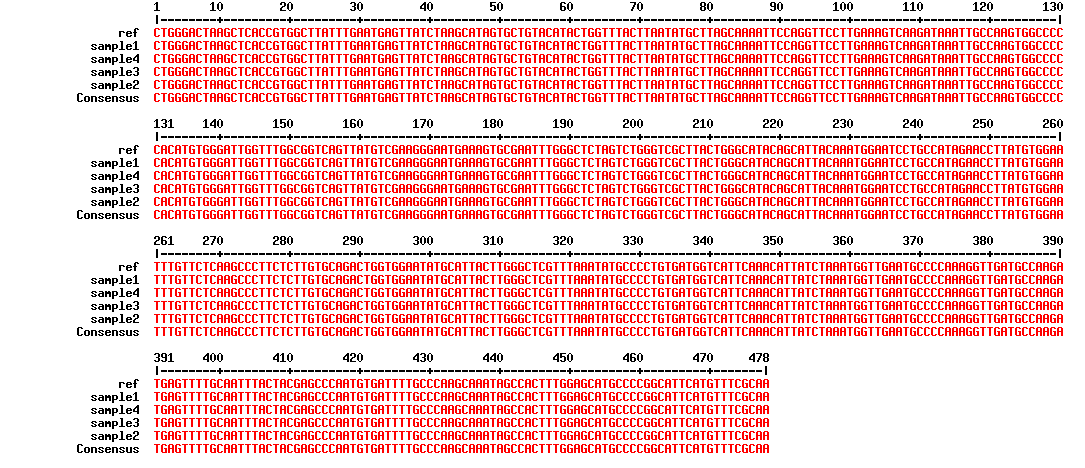

Supplement: Supplementary file 1 [file ijms-26-05946-s001.zip › Figure S2. Sequences alignment of RT-PCR products and the corresponding HTS-assembled genome region.gif]
